# Genetic and Clinical Features of Multiple Endocrine Neoplasia Types 1 and 2

**DOI:** 10.1155/2012/705036

**Published:** 2012-11-08

**Authors:** C. Romei, E. Pardi, F. Cetani, R. Elisei

**Affiliations:** Department of Endocrinology and Metabolism, University of Pisa, 56124 Pisa, Italy

## Abstract

Multiple endocrine neoplasia (MEN) are clinical inherited syndromes affecting different endocrine glands. Three different patterns of MEN syndromes can occur (MEN 1, MEN 2A, and MEN 2B). MEN syndromes are very rare, affect all ages and both sexes are equally affected. MEN 1 is characterized by the neoplastic transformation of the parathyroid glands, pancreatic islets, anterior pituitary, and gastrointestinal tract. Heterozygous *MEN 1* germline mutations have been detected in about 70–80% of patients with MEN 1. The mutations are scattered throughout the entire genomic sequence of the gene. MEN 1 patients are characterized by variable clinical features, thus suggesting the lack of a genotype-phenotype correlation. Therapeutical approaches are different according to the different endocrinopathies. The prognosis is generally good if adequate treatment is provided. In MEN 2 syndromes, the medullary thyroid cancer (MTC) is almost invariably present and can be associated with pheochromocytoma (PHEO) and/or multiple adenomatosis of parathyroid glands with hyperparathyroidism (PHPT). The different combination of the endocrine neoplasia gives origin to 3 syndromes: MEN 2A, MEN 2B, and FMTC. The clinical course of MTC varies considerably in the three syndromes. It is very aggressive in MEN 2B, almost indolent in the majority of patients with FMTC and with variable degrees of aggressiveness in patients with MEN 2A. Activating germline point mutations of the *RET* protooncogene are present in 98% of MEN 2 families. A strong genotype-phenotype correlation has been observed and a specific *RET* mutation may be responsible for a more or less aggressive clinical course. The treatment of choice for primary MTC is total thyroidectomy with central neck lymph nodes dissection. Nevertheless, 30% of MTC patients, especially in MEN 2B and 2A, are not cured by surgery. Recently, developed molecular therapeutics that target the *RET* pathway have shown very promising activity in clinical trials of patients with advanced MTC. MEN 2 prognosis is strictly dependent on the MTC aggressiveness and thus on the success of the initial treatment.

## 1. Introduction

The term multiple endocrine neoplasia (MEN) defines clinical inherited syndromes affecting different endocrine glands, each with its own characteristic pattern [1, 2]. In some cases, the tumors are malignant, in others, benign. Benign or malignant tumors of nonendocrine tissues occur as components of some of these tumor syndromes.

Three different patterns of MEN syndromes can occur (MEN 1, MEN 2A, and MEN 2B) with some new variants such as MEN 4, which is considered a variant of MEN 1 and the familial medullary thyroid cancer (FMTC), which is considered a variant of MEN 2A [3, 4]. These syndromes are familial and caused by inherited genetic mutations, which have been discovered within the last 20 years [5]. 

## 2. Multiple Endocrine Neoplasia Type 1

### 2.1. Definition

Multiple endocrine neoplasia type 1 syndrome (MEN 1, OMIM no. 131100), also known as Wermer's syndrome because of the description in 1954 by Dr. Paul Wermer of a pluriglandular dysfunction transmitted as a dominant trait, is characterized by simultaneous neoplastic transformation of multiple endocrine tissues, typically the parathyroid glands, pancreatic islets, and anterior pituitary. The case of an acromegalic patient with three enlarged parathyroid glands and a pituitary adenoma was indeed firstly described in 1903 and, after small case reports, Underdahl, Woolner and Black in 1953 described a series of 8 patients with various combinations of pituitary, parathyroid, and pancreatic islet adenomas [6].

**Figure 1:**
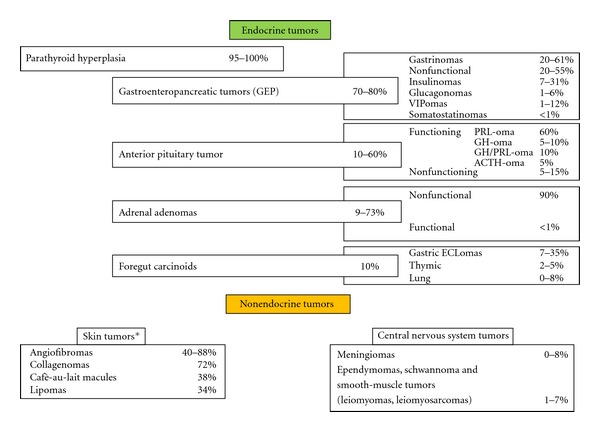
Prevalence of MEN 1 endocrine and nonendocrine manifestations. *Data obtained by a study on a series of 74 patients with MEN1 [8] and on a study determining the frequency of skin lesions in a series of 32 patients with MEN1 [9] Abbreviations: VIPoma-vasoactive intestinal peptide secreting tumor; PRLoma, prolactin secreting tumor; GHoma, growth hormone secreting tumor; ACTH, adrenocorticotropic hormone secreting tumor, ECLoma, enterochromaffin-like tumour.

This disorder is strongly suspected either in patients with endocrinopathies of at least 2 of the 3 main affected glands (i.e., parathyroid, enteropancreatic, and pituitary tumors) or in patients with at least one endocrinopathy in one of these organs and a first-degree relative who is affected by one of these tumors (familial MEN 1). Patients with features of MEN 1 syndrome but without a family history of MEN 1 are affected by a sporadic form of MEN 1. MEN 1 syndrome presents a wide spectrum of more than 20 endocrine and nonendocrine associated manifestations other than the classic endocrinopathies, including adrenocortical, gastric, thymic or bronchial tumors, foregut carcinoids, visceral and cutaneous lipomas, meningiomas, facial angiofibromas, concurring to different phenotypic presentations (Figure 1) [7–9]. Thyroid tumors are also frequently associated, but this association should be considered likely casual for the high incidence of thyroid abnormalities in the general population. Various clinical cases report rare combinations of less common tumors of MEN 1 and these atypical cases are also known as MEN 1 “phenocopy variants.”

The most frequent MEN 1-associated endocrinopathy, occurring in nearly 100% of patients by the age of 50 yrs, is primary hyperparathyroidism (PHPT), characterized by the synchronous or asynchronous development of multiglandular parathyroid hyperplasia with a benign course, while extremely rare is the occurrence of parathyroid carcinoma (PC), being only six cases of PC associated with MEN 1 in the literature [10]. Tumors of the parathyroid are often the first manifestation of MEN 1 in more than 85% of patients, with a typical age of onset of 20–25 yrs [11–13]. 

Gastroenteropancreatic endocrine (GEP) tumours, most arising in the pancreas as nonfunctioning neuroendocrine tumours or insulinomas, develop in up to 70–80% of MEN 1 patients, and gastrinoma represents, together with foregut carcinoids, the major cause of morbidity and mortality in MEN 1, because of its high rate of metastasis [17, 18] (Figure 1). The lesions range from microadenomas to macroadenomas, and to metastatic carcinomas. These tumors arise after the age of 40 yrs. Gastrinomas account for more than 50% of all GEP tumors and are typically small (<5 mm), multiple, mainly located in the duodenum and rarely in the pancreas. In the latter case it is difficult to distinguish these lesions from concomitant nonfunctioning pancreatic tumors (NFPTs).

The prevalence of pituitary tumors in MEN 1 ranges between 10% and 60%, being the prolactinoma the commonest MEN 1-related pituitary adenoma, although other pituitary tumors have been described so far (Figure 1). The majority of tumors are microadenomas (<10 mm). The mean age ± SD of onset has been reported to be 38 ± 15 yrs. Pituitary tumors are generally more invasive, symptomatic, with a higher prevalence of macroadenomas and a worse response to treatment than the sporadic counterparts [19, 20].

Foregut carcinoids, especially of the lung and thymus, are generally aggressive tumors and associated with a very high lethality. Adrenal tumors follow a benign course in most MEN 1 cases, and the majority are bilateral, hyperplastic, and nonfunctional [21]. Lipomas, both cutaneous and visceral, are present in about one-third of MEN 1 patients. Multiple facial angiofibromas occur in 40–80% of MEN 1 patients. Collagenomas are also common. These cutaneous lesions may be helpful for presymptomatic diagnosis of MEN 1 carriers.

### 2.2. Epidemiology

MEN 1 is rare, occurring in about one of 30,000 individuals, with an estimated prevalence of 2-3 per 100,000. The disorder affects all ages with a range of 5–81 yrs and both sexes equally [22]. A recent multicenter study analyzed 734 cases of MEN 1 and reported a different phenotype expression of the MEN 1 disease between males and females, in particular the prevalence of pancreatic tumors was higher in males than in females, while the opposite happened for the pituitary tumors. Thymic tumors were exclusively found in men. There was no significant gender difference in the prevalence and the probability of developing PHPT, adrenal and bronchial tumors in contrast to sporadic counterparts or in the proportion of positive genetic tests [23].

MEN 1-affected patients do not belong to particular geographical area, and there are no racial or ethnic preferences. No risk factors are known.

### 2.3. Pathogenesis

In 1988, linkage analysis studies in affected families placed the *MEN 1* gene within a 2 Mb interval in 11q13 and subsequently loss of heterozygosity [24] studies narrowed the location of the gene to a 600 kb interval [25]. The candidate gene, *MEN 1*, was finally identified by positional cloning in 1997 [26]. Combined LOH studies by microsatellite analysis in tumor tissues of MEN 1 patients and pedigree studies of large kindred supported a tumor suppressor function of the *MEN 1* gene suggesting the mechanism of biallelic inactivation firstly described by Knudson for the gene of retinoblastoma [27]. 

**Figure 2:**
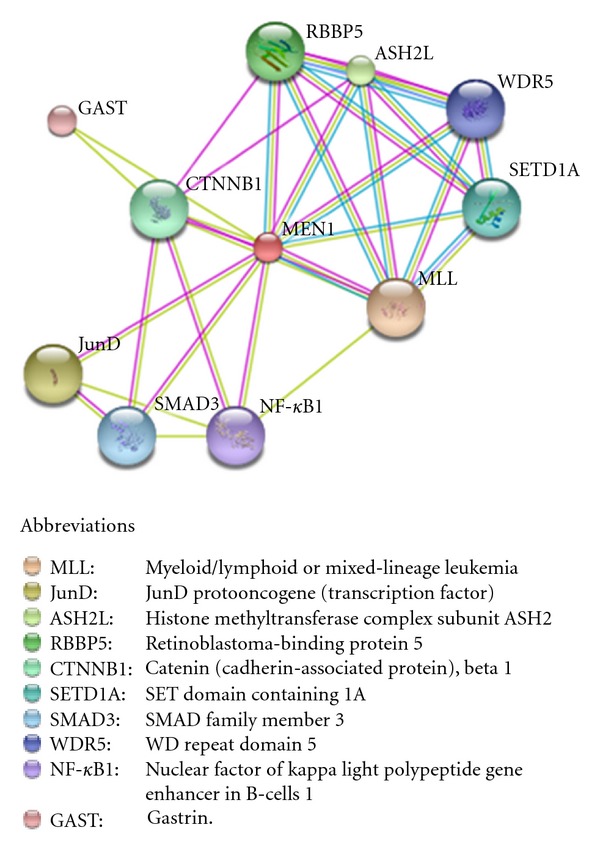
Predicted functional partners of menin generated by the protein interaction database STRING v.9.0. Evidences for these associations derive from experiments (pink lines), homology data (violet lines), predictions by text data mining (green lines), and information obtained from databases (light blue lines). The loss of one or more of these interactions might contribute to the development of MEN 1 syndrome by different mechanisms.

The *MEN 1* gene consists of 10 exons, the first of which is untranslated, spanning 7.2 kb of genomic sequence and encoding a protein, menin, of 610 amino acids, that does not present homologies to any other known proteins. *MEN 1* mRNA is expressed at a similar level in endocrine and nonendocrine organs, leaving unexplained the basis for endocrine predominance of neoplasia. Menin is a nuclear protein whose binding to the AP1 transcription factor JunD suggests a role in transcriptional regulation. The interaction with several partners and its participation in a variety of mechanisms, including regulation of cell proliferation and differentiation, apoptosis, endocrine/metabolic functions and the maintenance of genomic stability by DNA repair, have been so far reported [28]. The tumor suppressor nature of *MEN 1* gene is best achieved by menin-mediated inhibition of cell proliferation through multiple mechanisms such as (a) the interaction of menin with histone-modifying enzymes (MLL, EZH2, and HDACs) that affect gene transcription; (b) the interaction with various transcription factors, such as JunD, NF-*κ*B, PPAR*γ*, and VDR, to induce or suppress gene transcription; (c) the inhibition of cellular proliferation via TGF-*β* signaling and Wnt/*β*-catenin signaling pathways; (d) the repression of pro-proliferative factors (IGFBP-2, IGF2,s and PTHrP) involved in endocrine tumors; (e) the direct effect on cell cycle progression (Figure 2) [29]. The recent described crystal structure of the human menin should help us to better explain the opposite effects of the protein in the transcription process [30]. 

Heterozygous *MEN 1 *germline mutations have been detected in about 70–80% and 30% in patients with familial and sporadic MEN 1, respectively. The mutations are scattered throughout the entire genomic sequence of the gene, consistent with the lack of mutational hot spots. More than 1336 different germline and sporadic *MEN 1* gene mutations have been reported so far from the cloning of the gene [31]. More than 70% of *MEN 1* mutations lead to truncated form of the protein, confirming a loss-of-function mechanism.

To date, murine models of MEN 1 syndrome have been generated by disrupting different parts of the murine *MEN 1* gene localized on chromosome 19. The homozygous status shows a lethal phenotype at embryonic level, while the heterozygous mutant mice have a phenotype similar to the human MEN 1 disease, with a survival rate significantly lower than the wild-type mice, and with pancreatic islets lesions ranging from hyperplasia to insulin-producing islet cell tumors as the first manifestation [32]. Lesions of the parathyroid, pituitary, and adrenal glands occur later, and in addition to the typical MEN 1-associated endocrine tumors, these mice also develop tumors of the gonads and the thyroid. All the major tumors typically exhibit multistage tumor progression with metastatic potential [33, 34].

**Figure 3:**
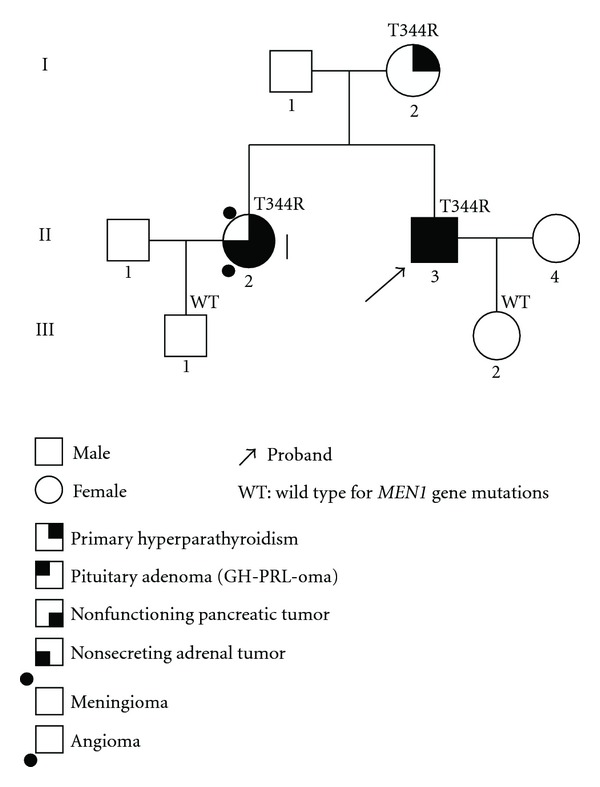
Pedigree of an Italian kindred positive for a germline MEN 1 missense mutation (T344R) in exon 10 (unpublished data). Generation numbers are represented by Roman numerals and individual numbers are in Arabic numerals. The proband, II-3, presents all the main MEN 1 endocrine manifestations; the sister, II-2, does not present pituitary lesions, but has nonendocrine lesions, such as a tumor of CNS and an angioma. Proband's mother, I-2, has only primary hyperparathyroidism, supporting the lack for a genotype-phenotype correlation.

The variable clinical expression between MEN 1 patients and relatives of the same family sharing the same genetic defect suggests the lack of a genotype-phenotype correlation (Figure 3) [35]. The lack of a correlation between the genetic status and the phenotypic expression could be due to either additional genetic events or epigenetic factors. A variant of the classic MEN 1 syndrome, known as MEN 1-Burin or “prolactinoma variant” of MEN 1, has a characteristic phenotype, such as a unusual higher incidence of carcinoid and pituitary tumors, all prolactinomas, a very low incidence of pancreatic endocrine tumors, and a late onset PHPT compared with families with typical MEN 1. Initially four large MEN 1-Burin kindreds were identified in the Canadian Newfoundland area and share a common nonsense mutation in the *MEN 1 *gene, suggesting the existence of a founder mutation [36]. Following the original report, similar families have also been described in Japan, Brazil, USA, and Mauritius carrying different nonsense or frameshift mutations suggesting that there is not a common *MEN 1* mutation in all MEN 1-Burin families [37, 38]. In addition, some kindreds may develop only PHPT, and this condition is referred to as familial isolated hyperparathyroidism (FIHP). Up to date mutations of the *MEN 1* gene, mostly missense, have been detected in 42 FIHP families [24, 39].

Approximately 20–30% of MEN 1 patients do not have *MEN 1* mutations, suggesting that other tumor susceptibility genes may be involved in the pathogenesis of this syndrome. A germline nonsense mutation in the human *CDKN1B* gene, encoding p27 protein, a negative regulator of cell cycle progression [40], has indeed been identified in a MEN 1 proband with acromegaly and PHPT, and a first-degree relative carrier with renal angiomyolipoma. The search for *CDKN1B* mutations in MEN 1 kindred started after the identification of a germline mutation of the *CDKN1B *gene in a rat colony affected by a variant of both MEN 1 and MEN 2 human syndromes, named MEN X [41]. This strain of rats developed multiple endocrine tumors, involving anterior pituitary adenoma, adrenal pheochromocytoma, thyroid C-cell hyperplasia, parathyroid and pancreatic islet cells hyperplasia. So far, germline mutations in the coding as well as in 5′ untranslated region of *CDKN1B* gene have been detected in other six MEN 1 kindred negative to *MEN 1* gene mutation testing [42, 43]. The predicted role in tumor predisposition of the* CDKN1B *mutations has been addressed with analyses *in vitro* and studies of protein localization and expression. This syndrome has been designated as MEN 4 (OMIM no. 610755).

### 2.4. Diagnosis

A clinical diagnosis of MEN 1 is made in individuals who have developed two or more of the classic MEN 1-associated tumors and in patients who have one classic MEN 1-related tumor and a family history of MEN 1. The biochemical diagnosis of PHPT, prolactinoma, and secreting endocrine tumors of the GEP tract in known or suspected MEN 1 is the same as for sporadic tumors (Table 1). Presymptomatic MEN 1 is biochemically detectable virtually one-two decades prior to full-blown phenotype, when symptoms are often related with the hormone hypersecretion or mass effect due to the growth of the tumor. Imaging studies on PHPT do not influence the indications for surgery [44]. Magnetic resonance imaging (MRI) is the test of choice for pituitary tumors [45]. Computed tomography (CT) and MRI are sensitive to detecting pancreatic endocrine tumors, adrenal, thymic, and lung carcinoids. Esophagogastroduodenoscopy with biopsy is recommended in patient with hypergastrinemia to detect peptic ulcer disease and carcinoids. In asymptomatic patients with MEN 1 endoscopic ultrasound (EUS) study is the most sensitive procedure to detect small (≤10 mm) pancreatic lesions [46]. For the identification of metastases of pancreatic tumors, the procedure of choice is the somatostatin receptor scintigraphy. The imaging test schedule of MEN 1-affected patients is summarized in Table 1 [3, 47]. 

#### 2.4.1. Genetic Testing

Mutation analysis of the *MEN 1 *gene may be used to confirm the clinical diagnosis, provide a genetic diagnosis in difficult cases, and screen asymptomatic relatives. The genetic testing of asymptomatic family members should be offered in early childhood since the first MEN 1 manifestations may occur by the age of 5 yrs [48]. *MEN 1* germline mutation testing should be offered to relatives of MEN 1 patients before biochemical and imaging screening examinations in order to exclude MEN 1 tumors. *MEN 1* gene testing can be helpful when clinical diagnosis is inconclusive; however but a suspicion of MEN 1 exists. The genetic analysis of the entire coding region and splice sites fails to detect *MEN 1* mutation in about 30% of typical MEN 1 kindred. If *MEN 1* mutation is not detected, testing for large gene deletions, haplotype analysis of *MEN 1* locus, or analysis of other genes should be considered [47]. 

#### 2.4.2. Screening Program of Tumor Expression in MEN 1 Gene Carriers

In contrast to the clinical importance of *RET* sequence testing in MEN 2, presymptomatic gene diagnosis has not been established to improve morbidity and mortality in MEN 1. Clinical practice guidelines on the management of *MEN 1* gene carriers who have not yet developed the disease have recently been published [47]. Briefly, annual biochemical screening should include the following measurements.

- PHPT: intact PTH and albumin-corrected total serum calcium or ionized serum calcium by age 8.
- Pituitary tumors: serum prolactin and insulin growth factor 1 (IGF-1) by age 5.
- Insulinoma: serum fasting glucose and insulin by age 5.
- Gastrinoma: gastrin, gastric acid output, and secreting stimulated gastrin.
- Other GEP tumors: proinsulin, glucagon, and plasma chromogranin A before the age of 10 yrs. Biochemical tests for adrenal lesions are not recommended unless the presence of symptoms or signs of functioning tumors and/or the detection of tumors with a diameter >10 mm on imaging.

Diagnostic imaging procedures are recommended for the identification of pituitary tumors (MRI every 3 yrs), GEP tumors (with the exception of gastrinoma and insulinoma) (MRI, CT, or EUS annually), adrenal lesions (MRI or CT annually), thymic and bronchial carcinoids (CT or MRI every 1-2 yrs).

### 2.5. Therapy

#### 2.5.1. PHPT

The optimal surgical approach is controversial. Approaches include either subtotal parathyroidectomy (PTx) (removal of 7/8 of the parathyroid tissue) with cryopreservation of parathyroid tissue, or total PTx and autologous parathyroid tissue graft in the forearm [49, 50].

At initial surgery, transcervical near total thymectomy is also recommended [3] since it may cure thymic carcinoids or prevent their development; in addition, the thymus is a common site for parathyroid tumors in MEN 1 patients with recurrent PHPT. Minimally invasive PTx is usually not recommended for the typical multiglandular involvement. Involvement of a highly experienced surgeon is crucial to optimal outcome. There are reports showing that the recurrence rate of PHPT in MEN 1 for procedures less than subtotal PTx were 8%, 31%, and 63% at 1, 5, and 10 years, respectively [51]. However, when subtotal or total PTx was performed, the rate of recurrence was 5%, 20%, and 39% at 1, 5, and 10 years, respectively. Rapid intraoperative PTH (iPTH) measurement can be helpful to prevent a persistent PHPT after glands removal [52]. Total PTx guided by iPTH monitoring and followed by autograft to the forearm led to a 10% of recurrences in the autografted parathyroid after a mean time of years after surgery [53].

#### 2.5.2. GEP Tumors

**Gastrinoma**

The therapy in MEN 1-associated gastrinoma aims for the treatment of acid hypersecretion and the resection of the tumor [54]. However, surgical versus nonsurgical management of gastrinoma in MEN 1 syndrome is still controversial since successful outcome of surgery is rare. When surgery is not possible, the medical treatment may include somatostatin analogs, interferon-alpha, and chemotherapy. Proton pump inhibitors or H2-receptor blockers are able to reduce gastric acid output in these patients.

**Other GEP Tumors**

The surgical approach for asymptomatic NFPT in MEN 1 is controversial. The choice between a preserving pancreatic-duodenectomy or a more aggressive approach depends on the estimated risk for the development of metastatic disease, the size of the lesions, and the functioning nature of the tumour [55]. Surgery is usually indicated for insulinoma. Somatostatin analogs, radionuclide therapy, biotherapy, and chemotherapy may be used in inoperable tumors [47]. In cases of inoperable or metastatic well-differentiated tumors, sunitinib or everolimus may be considered [56].

#### 2.5.3. Pituitary Tumors

Treatment of pituitary tumors in MEN 1 is identical to that in sporadic tumors. Dopamine agonists, especially cabergoline, are the preferred treatment of PRL-secreting tumors. Transsphenoidal surgery is the treatment of choice in GH-secreting tumors with a success rate of 50–70%. Somatostatin analogs (octreotide and lanreotide) are considered the current medical treatment of choice of GH-secreting tumors and are able to normalize the serum levels of GH and IgF1 in ≥50% of patients. Dopamine agonists can be used in mixed GH-PRL secreting tumors and in cases of tumors resistant to somatostatin analogs. Surgery is the treatment of choice in ACTH-secreting pituitary tumors. Radiation therapy can be used in cases of persistent or recurrent disease.

#### 2.5.4. Adrenal Tumors

Treatment of adrenal tumors in MEN 1 is similar to that for sporadic tumor. Surgery is the treatment of choice in functioning tumors and nonfunctioning tumors with significant growth over a 6-month interval, suspicious radiological features, and greater than 4 cmin size [57].

#### 2.5.5. Thymic, Lung, and Gastric Neuroendocrine Tumors

The treatment of choice for thymic and lung carcinoids is surgery. When surgery is not possible, chemotherapy and radiotherapy should be considered.

The optimal therapy of gastric carcinoids is controversial. Endoscopic excision or partial/total gastrectomy is required for tumors >10 mm. Lesions <10 mm can be monitored by endoscopy [47]. 

### 2.6. Prognosis

The prognosis is generally good if adequate treatment is provided for parathyroid, pancreatic, and pituitary tumors. Pancreatic endocrine tumours associated with MEN 1 are less malignant than sporadic tumors and carry a better prognosis, with a median survival of 15 years compared to 5 years for patients with sporadic tumors. This may reflect more indolent disease or earlier diagnosis [58].

## 3. Multiple Endocrine Neoplasia Type 2

### 3.1. Definition

Multiple endocrine neoplasia type 2 syndrome (MEN 2) is characterized by the association of benign and malignant endocrine neoplasia with other nonendocrine diseases. In all syndromes, the medullary thyroid cancer (MTC), originating from C cells is present and can be associated with pheochromocytoma (PHEO) and/or multiple adenomatosis of parathyroid glands with hyperparathyroidism (PHPT). The different combination of the endocrine neoplasia with or without nonendocrine diseases gives origin to 3 different syndromes: MEN 2A, MEN 2B, and FMTC, this latter being considered as a variant of MEN 2A. 

Although MEN 2 was firstly detected in the 19th century at the University Hospital of Freiburg, Germany [59], the association of an MTC and an PHEO in a single patient (Sipple's syndrome) was firstly described in 1961 [60, 61]. However, the entire entity of MEN 2A was recognized only in 1968 in a family with PHEO, MTC, PHPT, and Cushing's disease [62].

MEN 2A (OMIM 171400) syndrome is the most common form. Almost all affected patients develop MTC which is usually multifocal, bilateral and almost invariably associated with C-cells hyperplasia. Fifty percent of MEN 2A patients are at risk of developing PHEO which, although frequently asynchronous, is usually involving both adrenal glands. About 25% of MEN 2A patients can also develop PHPT [63]. MTC is generally the first manifestation of MEN2A and develops between the ages of 5 to 25 years [16]. PHEO usually presents after MTC or concomitantly; however, it has been reported as the first sign of the syndrome in 13–27% of MEN 2A cases [64, 65]. In some cases, Hirschsprung' s disease (HSCR) [66, 67], a congenital disease characterized by the aganglionosis of the gut and/or cutaneous lichen amyloidosis [68–70], a pruritic lichenoid skin lesion usually located in the interscapular region, is associated with MEN 2A (Table 2).

MEN 2B syndrome (OMIM 162300) is the least common but the most aggressive form of MEN 2 (5–10% of all cases) [71]. Patients rarely become adults since the metastatic lesions of MTC develop and progress very rapidly. In MEN 2B patients, MTC is associated with PHEO in 45–50% of cases, while an association with PHPT was never described. Typically, almost 100% of MEN 2B patients develop mucosal neuromas, bumpy lips, ganglioneuromatosis of the gastrointestinal tract, and a Marfanoid habitus [72] (Table 2).

Familial MTC (FMTC; OMIM 155240) is considered the mildest variant of MEN 2 since in patients with FMTC there is a strong predisposition to develop MTC but a very low incidence of the other clinical manifestations of MEN 2A [73]. It has been diagnosed more frequently in recent years (35–40% of all cases), and particularly after the introduction of the genetic test [74, 75]. The clinical diagnosis of FMTC can only be posed when four or more family members across at least 2 or more generations have isolated MTC [3, 4, 68]. In the absence of these criteria, to prove that a subject has an FMTC, it is necessary to demonstrate the presence of a germline *RET* mutation [3]. Whereas MEN 2A and 2B are clinically very well defined, the lack of specific clinical features and/or familial history makes the diagnosis of FMTC relatively difficult, thus generating an underestimation of FMTC prevalence within families, especially in series where no genetic test for *RET* mutation has been performed. From the discovery of the first kindred affected by MTC, it was clear that these syndromes are inherited with an autosomal-dominant mendelian mechanism. For this reason, 50% of first-degree relatives of the index case (i.e., parents, siblings, and children) may be affected.

### 3.2. Epidemiology

MEN 2 syndrome is a very rare disease. To have a better idea of the rarity of the disease, one can consider that MEN 2 syndrome represents 25% of all MTC cases and that MTC represents only 5–10% of all thyroid malignancies, which represent only 1% of all human malignancies. Thus, the overall prevalence of MEN 2 syndromes is very low, accounting for about 0.02-0.03% of all human tumors. The total prevalence of all MEN2 variants has been estimated approximately 1/30,000 individuals [4].

**Figure 4:**
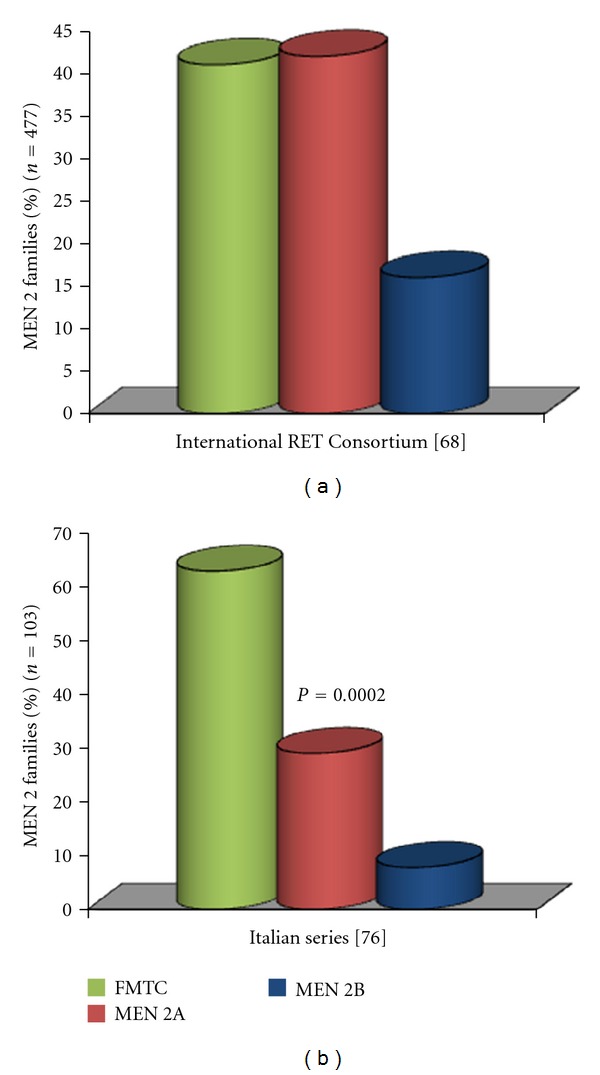
Prevalence rates of the three MEN 2 phenotypes in the International RET Consortium series (a) and in an Italian series (b). A higher prevalence of the FMTC phenotype was observed in the Italian series with respect to that reported by the International Consortium, which was based on cases collected up to 1994-1995.

The relative prevalence of the 3 syndromes reported in the first International *RET* consortium in 1994 [68] (Figure 4(a)) was significantly different from that reported in more recent studies [76] (Figure 4(b)). In particular, this change has been observed after the introduction of the *RET* genetic screening which allowed to recognize several cases of hidden FMTC.

### 3.3. Clinical Manifestation

The clinical appearance of MTC in MEN 2 syndromes is that of a thyroid nodular disease, similar to that of the sporadic form with the exception that it is usually bilateral, multicentric, and associated with C cell hyperplasia, which is considered a preneoplastic lesion. The clinical course of MTC varies considerably in the three syndromes. It is very aggressive and almost invariably unfavourable in MEN 2B, with affected patients rarely surviving after the adolescence. It is almost indolent in the majority of patients with the FMTC and shows variable degrees of aggressiveness in patients with MEN 2A. It is the only malignant tumor and the most severe disease of the syndrome so that in the majority of cases the prognosis of the disease is mainly related with the prognosis of the MTC.

An age-related progression to MTC has been described with younger age of onset for MEN 2B (youngest reported 0.6 year), older age for FMTC (usually adult age > 20 years), and intermediate age (starting from 1.5 years, but childhood age is the most prevalent) [16] (Table 3).

Up to 70% of MTC patients have already cervical lymph node metastases at the diagnosis [77] and this is a unfavorable prognostic factor for the cure of the disease. About 30%, mainly belonging to MEN 2B and, to a lesser extent, to MEN 2A, have already distant metastasis at the time of diagnosis and this is an unfavorable prognostic factor for the survival although they have a median survival of 5–10 years. 

MTC is usually the first neoplastic manifestation in most MEN2 kindred because of its earlier and overall higher penetrance. With few exceptions, PHEO and PHPT are usually discovered few years after the MTC diagnosis. Both PHEO and PHPT are benign diseases, but, when present, they can severely affect the patient with severe hypertension or unexpected hypertensive crisis and hypercalcemia, respectively.

Both CLA and mucosal and/or corneal nerves neurinomas associated with a Marfanoid habitus are strongly suggestive of MEN 2A or MEN 2B, respectively. 

### 3.4. Pathogenesis

During the 80s, genetic linkage analysis localized the MEN 2 gene into the centromeric region of chromosome 10. In 1993, *RET* germline mutations were recognized as the causative molecular alterations in MEN 2 syndromes [78–80]. The *RET* protooncogene is a 21-exon gene and encodes for a tyrosine kinase transmembrane receptor located on chromosome 10q11.2. The receptor is composed of an extracellular domain (EC), with a distal cadherin-like region and a juxtamembrane cystein-rich region, a transmembrane domain (TM) and an intracellular domain with tyroisine-kinase activity (TK). In physiological conditions, the activation of the *ret *protein is secondary to its dimerization due to the interaction with one of its ligands. Four different ligands have so far been recognized: the glial cell-line derived neutrophilic factor (GDNF), neurturin (NTN), persepin (PNS) and artemin (ART). The interaction is mediated by a ligand-specific coreceptor (e.g., the GFR*α*-1 is the co-receptor for the GDNF). The dimerization of *ret* protein induces the autophosphorylation of the TK domain and the activation of downstream signaling pathways.

Activating germline point mutations of the RET proto-oncogene are causative events in MEN 2A, MEN 2B, and FMTC. RET mutations have been found to be widely distributed not only among the 5 cysteine codons 609, 611, 618, 620, and 634 but also in other noncysteine codons, such as codon 804 in exon 14, codon 883 in exon 15, and others. These widely spread non cysteine mutations are mainly associated with FMTC phenotype [74–76]. Virtually, all the mutations reported up to now are present on public databases (http://www.hgmd.cf.ac.uk; http://www.arup.utah.edu/database/MEN2). Their prevalence, which is clearly different in different countries [14–16], is reported in Table 4.

After the introduction of genetic screening in the diagnostic procedures of patients affected with apparently sporadic MTC, new mutations were found, especially in noncysteine-rich regions [74, 81, 82], that were mainly associated with FMTC [76]. Sometimes these new mutations are very rare, present only in a few families and a few family members, raising doubts as to whether they represent the driving force of the tumoral disease or result from the genetic screening associated with MTC [83, 84]. 

Apart from genetic alterations, no risk factors have been associated with the development of MEN 2 syndrome.

### 3.5. Genotype-Phenotype Correlation

The MEN 2 syndromes are characterized by a strong genotype-phenotype correlation and a specific RET mutation may be responsible for a particular phenotype and a more or less aggressive clinical course. This close association was firstly identified in an early study of 477 families affected by MEN 2 [68] and confirmed by several other studies. This correlation can be summarized as follows.

- approximately 98% of families with MEN 2A have a germline RET mutation in exon 10 or 11 [4, 68, 74]. Mutations at codon 634 (exon 11) is the most frequently found in typical MEN 2A families (87%): in this case the 3 endocrinopathies (i.e., MTC, PHEO and PHPT) are usually present both in the same subject and in several family members; mutations of cysteine residues at codons 609, 611, 618, and 620 are usually present in the other MEN 2A cases in which the combination of the 3 endocrinopaties is less common [4, 85, 86];
- germline *RET* mutations are found in approximately 95% of families with FMTC [76, 85]. These mutations are mainly affecting the non cysteine codons located at exons 5, 8, 13, 14 and 15 with 20% to 30% of mutations located at one of the five cysteine residues (codons 609, 611, 618, 620, and 634). A different geographic distribution has been reported especially for cysteine and non cysteine mutations [15, 16, 76] (Table 4);
- about 95% of individuals with the MEN 2B phenotype have a single point mutation in the tyrosine kinase domain of the *RET* gene at codon 918 in exon 16, which substitutes a threonine for methionine (M918T) [68]. Another mutation at codon 883 in exon 15, A883F, has been identified in several affected individuals without a M918T mutation. Tandem *RET* mutations of codons 805, 806, and 904 in cis configuration with the V804M mutation have also been reported in individuals with MEN 2B [87, 88]. Taken together, *RET* mutations have been found in more than 98% of individuals with MEN 2B.

The genotype-phenotype correlation clearly indicates that not all mutations confer the same aggressiveness to MTC. A similar evidence is for the different levels of disease penetrance. The American Thyroid Association recently categorized the *RET* mutations into four levels of risk (Table 5); these levels are of great usefulness for the identification of the therapeutic and follow-up strategies [4]. 

### 3.6. Genetic Testing

All patients affected by MTC, both those with a familial history of MEN 2 and those with an apparently sporadic form, must undergo a germline *RET* protooncogene analysis. The major reason to test apparently sporadic MTC is the evidence that 5–10% of these cases are indeed “hereditary” cases since they harbor a germline *RET* mutation [89].

When a germline mutation is found, all first-degree relatives should be submitted to *RET* analysis to distinguish “gene carriers” from “nongene carriers.” The *RET* gene carriers are at very high risk to develop MTC and they must be submitted to a diagnostic and therapeutic strategy which is very much conditioned by the ATA level of risk of the mutation. Recently, a greater importance has been recognized to serum calcitonin measurement for planning the timing of thyroidectomy which should be either prophylactic or very precocious when the tumor is still intrathyroid [90]. In nongene carriers the risk to develop MTC is similar to that of the general population and they should not be submitted to any further specific test.

The genetic screening activity should be accompanied by genetic counseling that should involve specific figures such as the geneticist, who will explain the particular type of transmissibility of the disease, the endocrinologist, who will explain the particular type of pathology and the risk of developing the different endocrine disorders, and possibly a psychologist to address issues arising from the knowledge of being a “gene carrier.”

As stated above, the identification of the type of mutation also gives information about the possible phenotype suggesting the diagnostic and therapeutic strategy to be followed. Although all cases of hereditary MTC should be evaluated for the possibility of developing PHEO or PHPT, some of them are more likely to manifest these diseases while others will never develop them or in a late stage of the disease. 

### 3.7. Clinical Diagnosis

Clinical evaluation of MEN 2 patients consists in the measurement of basal and/or pentagastrin-(Pg-) stimulated serum calcitonin (CT), neck ultrasound, and fine needle aspiration of thyroid nodule if present. To rule out the presence of an PHEO, an abdominal ultrasound should be performed accompanied by the measurement of both plasmatic and urinary epinephrine and norepinephrine; whenever possible, the measurement of metanephrines is better recommended for their higher sensitivity. Serum PTH, calcium, and vitamin D measurement should be always performed for the diagnosis of PHPT. The physical examination of these patients is also important particularly in MEN 2B syndrome because the phenotype is quite typical being characterized by Marfanoid habitus, mucosal and/or corneal nerves neurinomas. The presence of an itchy/dark spot in the interscapular region should rise the question of a possible CLA that is highly suggestive of MEN 2A.

### 3.8. Conventional Therapy

The treatment of choice for primary MTC, both sporadic or hereditary, is total thyroidectomy with systematic dissection of all lymph nodes of the central compartment. Total thyroidectomy is necessary as MTC is multicentric in 65–90% of patients in MEN 2 and extensive central lymph node dissection has been reported to improve survival and recurrence rates compared to less aggressive procedures [91, 92]. Lymph node dissection of laterocervical compartments is not performed on principle but only when the neck ultrasound suggests the presence of metastatic nodes. 

Endoscopic adrenal-sparing surgery has become the method of choice for the surgical therapy of PHEO [93]. In cases with an asynchronous development of PHEO, the adrenal gland without PHEO can be preserved, but the patient must be aware that the probability to repeat the surgical treatment in the near future is very high. The advantage of a monolateral adrenal surgery is the possibility to avoid substitutive therapy until the second surgery will be performed. 

The parathyroid glands are frequently found to be enlarged at the time of the thyroidectomy for MTC and should, therefore, be carefully evaluated. The goal in MEN 2 patients with PHPT is to excise the enlarged glands and to leave at least one apparently normal parathyroid gland intact. If all glands are enlarged, a subtotal parathyroidectomy or total parathyroidectomy with autotransplantation should be performed. In patients with persistent or recurrent PHPT, the long-term oral administration of calcimimetic drugs as cinacalcet to achieve long-term reductions in serum calcium and PTH concentration should be considered.

### 3.9. Prophylactic or Precocious Thyroidectomy in *RET* Gene Carrier

Prophylactic thyroidectomy is advised in gene carriers to guarantee a definitive cure in these subjects. Four different risk levels (from A, the lowest, to D the highest) for *RET* mutations have been suggested by the American Thyroid Association task force, which developed the most recent guidelines for the management of MTC patients [4]. According to these guidelines, these levels of risk, which are related to the clinical aggressiveness of the corresponding MTC, should be taken into consideration when planning surgical treatment. In particular patients with a level D, *RET* mutation (i.e., Met918Thr) should be treated as soon as possible in the first year of life; patients with level B and C mutations (located in exons 10, 11, 13, 14, and 15) should be operated on before 5 years of age; only for patients with a level A mutation (exon 8 and 5 mutations), total thyroidectomy can be delayed after five years of age or until the CT positivity. 

Recently, some evidences in big series of *RET* gene carriers demonstrated that gene carriers with undetectable levels of basal CT have an almost null risk to have already developed the MTC [90, 94, 95]. Moreover, a serum Ct <30–40 pg/mL is always associated to an intrathyroidal micro-MTC without any evidence of lymph node metastases. Taking into account these observation, Elisei et al. [90] designed a study in which they operated on only *RET* gene carriers on the basis of basal and stimulated CT. According to their results, the time of surgical treatment could be personalized and safely planned when the stimulated serum CT becomes positive at the annual control, independently from the type of *RET* mutation and its associated level of risk. Of course, both cysteine *RET* mutations and older age are risk factors for having an earlier positive result for either basal or Pg-stimulated serum CT. For these reasons, the follow-up controls should be more or less frequent in cysteine or noncysteine *RET-*mutated gene carriers, respectively. This strategy obviously implies a high compliance of the *RET* gene carriers to the scheduled followup with the advantage that young children can be treated later, sometime even after the puberty, close to the adulthood. 

### 3.10. Target Therapy for Persistent MTC

Thirty percent of MTC patients, especially in MEN 2B and 2A, are not cured by surgery. They remain affected and can develop, if not already present at the time of the diagnosis, distant metastasis in the lungs, liver, bone and, more rarely, brain. Several studies demonstrated that conventional therapies, such as chemotherapy and radiotherapy, did not determine any clinical benefit [96, 97]. Until few years ago, patients with advanced and progressive MTC were “orphan” of drugs. Recently, developed molecular therapeutics that target the *RET* pathway have shown very promising activity in clinical trials of patients with advanced MTC [98]. In the majority of cases, the drug is a multityrosine kinase inhibitor (TKI) with the ability to block not only *ret *butalsoone or more of the vascular endothelial growth factor receptors (VEGF-R) as well as C-MET and/or C-KIT or FLT3 and/or other kinases. Vandetanib has been recently approved both by FDA (Food and Drug Administration) and EMA (European Medical Agency) for the treatment of advanced and progressive MTC. Other TKIs, such as sorafenib, sunitinib, motesanib, lenvatinib, AND cabozantinib, are still under investigation either in official phase II/III clinical trials or in “off-label” studies [99]. Although very promising, further studies and longer followup are needed to better evaluate the clinical benefits in terms of progression-free survival and overall survival as compared to the discomfort determined by the side effects which is not negligible. Among several, the most severe and intolerable side effects are anorexia, weight loss, and fatigue, which are difficult to be controlled. Others, such as hypertension or skin lesions can be managed with standard care procedures. A list of drugs used in ongoing clinical trials is reported in Table 6.

## 4. Conclusions

MEN syndromes are genetic disease transmitted with an autosomal dominant trait. Although rare, they caught the attention of both endocrinologists and geneticists and much information has been collected in the last decades. We know the genetic alterations of both MEN 1 and MEN 2, how they are transmitted, their prevalence, and the relationship between genotype and phenotype. Much is also known about clinical features and possible treatments. Despite all, information still remain to discover the genetic of MEN cases who are orphan of *MEN 1* or *RET *genes germline mutations.

## Figures and Tables

**Table 1:** Biochemical and imaging screening for tumors in MEN 1 patients.

Tumor	Annual biochemical tests	Imaging tests
Parathyroid	Calcium, PTH	—
GEP	Gastrin, glucagon, vasointestinal polypeptide, pancreatic polypeptide, chromogranin A, insulin, fasting glucose	MRI, CT, and endoscopic ultrasound (annually), gastroscopy with biopsy every 3 yr in patients with hypergastrinemia
Pituitary	PRL, IgF1	MRI (every 3–5 yrs)
Adrenal	—^1^	CT or MRI (every 3 yrs)
Thymus and lung	—	CT or MRI (every 1-2 yrs)

^1^Biochemical test should be performed in patients with tumors greater than 1 cm or with clinical features.

**Table 2:** Prevalence of tumoral diseases in MEN 2 syndromes.

Phenotype	MTC (%)	PHEO (%)	HPT (%)	Nonendocrinological associated pathologies (%)
MEN 2A	95	50	25	Cutaneous lichen amyloidosis (10%)
				Hirschsprung's disease (2%)
MEN 2B	95	50		Mucosal neuromas (100%)
				Marfanoid habitus (100%)
				Ganglioneuromatosis of the gastrointestinal tract (60%)
FMTC	100			

**Table 3:** Age of onset of endocrine tumors according to RET Mutation MEN 2 database. Data from ARUP Scientific Resource for Research and Education.

RET codon	MTC (years)	PHEO (years)	HPT (years)
533	21	34	
609	4	19 (C609S)	38
611	6	30	40
618	5	19	41
620	5	19	
630	1		32
634	0.8	5	10
768	9	59	
790	10	28	
791	15	38	
804	6	28	9
883	10		
891	9	46	17
918	0.17	12	

**Table 4:** Different prevalence of RET germline mutations in hereditary MTC in different European countries.

RET mutation	Italy (*n* = 246) [14]	Germany (*n* = 141) [15]	Euromen (*n* = 145) [16]
Cys634	86 (34.9%)	57 (40%)	98 (67.6%)
Val804	52 (21.1%)	9 (6.4%)	3 (2.1%)
Ser891	23 (9.3%)	3 (2.2%)	3 (2.1%)
Met918	20 (8.1%)	21 (15%)	4 (2.8%)
Cys618	15 (6.0%)	7 (5%)	10 (6.9%)
Glu768	9 (3.6%)	2 (1.4%)	1 (0.8%)
Cys620	9 (3.6%)	10 (7%)	10 (6.9%)
Leu790	8 (3.2%)	17 (12%)	7 (4.8%)
Cys609	6 (2.4%)	1 (0.7%)	1 (0.8%)
Cys630	4 (1.6%)	1 (0.7%)	1 (0.8%)
Cys611	1 (0.4%)	2 (1.4%)	4 (2.8%)
Tyr791	1 (0.4%)	10 (7%)	3 (2.1%)
Ala883	1 (0.4%)	0	0
Cys515	1 (0.4%)	0	0
Lys666	1 (0.4%)	0	0
Met848	1 (0.4%)	0	0
Ser904	1 (0.4%)	0	0
Thr338	1 (0.4%)	0	0
Asp631	0	1 (0.7%)	0
No mutations	6 (2.4%)	0	0

**Table 5:** Classification of RET mutations according to ATA risk level.

ATA risk level	RET codons
A (low)	768, 790, 791, 804, 649, 891
B (medium)	609, 611, 618, 620, 630, 631
C (high)	634
D (highest)	918, 883

**Table 6:** Drugs used in ongoing clinical trials for the treatment of advanced MTC and other thyroid tumors.

Drug	Molecular target
Axitinib	VEGFR, PDGFR*β*, C. Kit
Gefitinib	EGFR
Imatinib	VEGFR, RET, BCR-ABL
Motesanib	VEGFR, RET, PDGFR*β*, C. Kit
Sorafenib	VEGFR, RET, RET/PTC, BRAF, PDGFR*β*, C. Kit
Sunitinib	VEGFR, RET, RET/PTC, PDGFR*β*
Vandetanib	VEGFR, RET, RET/PTC, EGFR
XL184	VEGFR, RET, PDGFR*β*
